# Mycoplasmas and Human prostate cancer: An exciting but cautionary note

**DOI:** 10.18632/oncotarget.282

**Published:** 2011-06-01

**Authors:** Shyh-Ching Lo, Shien Tsai

**Affiliations:** Tissue Microbiology Laboratory, Division of Cellular and Gene Therapies, Office of Cellular, Tissue and Gene Therapy, Center for Biologics Evaluation and Research, Food and Drug Administration, Bethesda, MD 20892

A recent study of Barykova, *et al*. [1] described a significant association between *Mycoplasma hominis* infection and development of prostate cancer. In the study, the authors detected the presence of *M. hominis*-specific DNA sequences in examining 496 prostate biopsies by PCR obtained from 248 Russian men undergoing cancer diagnosis. The association appeared to be particularly significant in the statistical analysis between *M. hominis* infection and development of the higher grade prostate cancer. A possible role for mycoplasmas in association with human malignancy was first noted during 1960s. Several studies reported isolation of mycoplasmas from human leukemic bone marrow [2-6]. A majority of the mycoplasmas isolated in the studies were identified as *Mycoplasma fermentans*. However, the mycoplasma-oncogenesis hypothesis failed to advance because the same mycoplasmas later could also be found in non-leukemic children or adults, although the mycoplasmas appeared to be most frequently isolated from patients with leukemia [7]. Koch's postulate of identifying the causative agent in an infectious disease by finding a specific pathogen uniquely present in a particular disease process overshadowed the characteristics in both chronicity and multi-factorial nature of the malignant process.

Mycoplasmas are a heterogeneous group of very small organisms capable of self-replication. The wall-free microbes can cause a wide variety of diseases in animals. Some mycoplasmas cause respiratory or urogenital diseases in humans. However, many other mycoplasmas chronically colonize our respiratory and urogenital tracts without apparent clinical significance [8]. Various mechanisms including “antigenic variation” may be critical in the ability of these microbial species to evade host antibody responses or other elements of immune recognition [9]. Most interestingly, the wall-free mycoplasmas are among the few prokaryotes that can grow essentially “symbiotically” in cultures of mammalian host cells. Mycoplasmas are often growing in a close interaction with mammalian cells for a very long period of time without producing detectable cytopathic effects on the infected host cells. Thus, their infections in cell cultures are commonly unrecognized. However, we believe the interplay between human hosts and these unusual microbes is complex and can be important in the development of various human chronic illnesses including cancers. Our laboratory at the Armed Forces Institute of Pathology (AFIP) had long been interested in unusual infections of mycoplasmas. We have previously hypothesized chronic infections by the seemingly low virulent microbes could produce a previously unrecognized form of pathogenesis such as neoplasia in the infected mammalian hosts [10].

Over the years, we developed several *in vitro* mycoplasma-oncogenesis models to demonstrate that infections of mycoplasmas could not only damage DNA or genes of infected mammalian cells but also disturb the cell cycle check points that control normal cell division and the process of apoptosis by providing potent altered cell growth signals [11]. Various signals could continually be sent through close interacting with mycoplasmas from the surface of chronically infected host cells to alter the expression of many different genes, thus significantly affect crucial biological characteristics including control of cell growth [10, 11]. Thus, we hypothesized that chronic infections by mycoplasmas produced genetic instability and chromosomal aberrations in the mycoplasma-infected cells that however failed to undergo proper apoptosis due to mycoplasma-mediated mitogenic and anti-apoptotic effects would gradually lead to the consequence of malignant transformation [10-12]. Studies of *in vitro* model systems, which are confined to a fixed condition and may not describe the full range of activities occurring in the infected host, could nevertheless provide valuable information on mechanisms of malignant cell transformation. We believe the chronic persistent infection models of mycoplasmas developed have provided an excellent *in vitro* model system for cancer research. Most importantly, the *in vitro* mycoplasma-oncogenesis models developed have a long latency with a multi-stage malignant progression, one of the most common paradigms in naturally occurring human cancers [13].

Mycoplasmas are ubiquitously colonizing in both respiratory and urogenital tracts of human body. The most frequently identified Mycoplasma species in human urogenital tract are *M. hominis, Ureaplasma urealyticum and M. genitalium*. Naturally, one would suspect that some of these urogenital mycoplasmas could also infect or colonize prostate and play an important role in the prostate disease processes like hyperplasia or cancer. In the present publication of Barykova, *et al*. [1], the results showed a statistically significant association between *M. hominis* infection and development of prostate cancer. This study in Russian men is the first report demonstrating the clinical association between *M. hominis* infection in prostate and prostate cancer formation. The significant association could be found in PCR assay of detecting *M. hominis-* specific DNA, 16S r-RNA and IgG serology studies. The presence of *M. hominis* in some of the prostate biopsies (6.3%), although at a lower frequency, could be confirmed by isolation of the mycoplasma by culture. However, the culture isolation and characterization of *M. hominis* from prostate biopsies with or without pathology should be further studied with great care in the future to confirm the association. In a similar note, since many men may have *M. hominis* colonization in the urogenital tract as commensal, the background of anti-*M. hominis* IgG level was found to be high and significantly varying with men's age. Interestingly, we previously reported men older than 60 showed marked decrease of *M. hominis* infection by serology [14)]. Large-scale epidemiological studies as proposed to be conducted by authors of the article will indeed be required to demonstrate the association between *M. hominis* infection and prostate cancer contracted mainly by older males. Other urogenital mycoplasmas such as *M. genitalium* should also be further examined for their potential involvement in the prostate disease process. Parallel studies of other human uogenital mycoplasmas could certainly serve as an important control for *M. hominis*.

In addition to large-scale epidemiological studies proposed by Barykova *et al.* to confirm the significance of their observations, it also would be essential to develop an *in vitro* cell culture model as well as an animal model for further evaluation of the role of *M. hominis* in the development and progression of prostate carcinogenesis. In this context, Namiki *et al*. [15] recently reported that chronic exposure of the immortalized human benign prostate cell line, BPH-1 to *M. hyorhinis* or *M. genitalium* could induce malignant transformation of the prostate epithelial cells. The prolonged mycoplasma-infected BPH-1 cells evidently gained the critical malignant property of forming tumor when introduced into nude mice. We expect that chronic infection with *M. hominis* would also induce BPH-1 cells to undergo malignant transformation, as demonstrated by the infections of *M. hyorhinis* and *M. genitalium*. However, it may be important to note that the immortalized human BPH-1 cell line was previously established by transduction of SV40 T-antigen into primary cultures of prostatic epithelial cells. The BPH-1 cells expressing the viral antigen might be more susceptible to malignant transformation when exposed to the appropriate stimulation such as mycoplasma infections. Therefore, the role of mycoplasma infection played in the BPH-1 cell culture model could be related to the promotion of host cell to undergo further progression to a more malignant phenotype, rather than an initiation of cell transformation (15). Using human PBMCs in the cultures that normally died quickly in the first few weeks, our previous study showed introduction of *M. fermentans* (Mi and PG18 strains) into the PBMC cultures would markedly enhance the blood cells to undergo immortalization [16]. Since all the immortalized lymphocytes obtained after many months were found to be positive for EBV latent membrane protein (LMP1) antigen, the mycoplasma-mediated effect in blood cell immortalization could also be promotional effect, instead of initiation effect.

It has long been documented in both clinical and pathological studies that majority of men older than 50 years have various form/degree of hyperplasia and a very significant proportion (~50%) among them already harbor prostate cancer. However, only a small percent of the prostate cancer will continue to progress into highly malignant form and undergo aggressive metastasis. Factor(s) that could be having significant promotional effect in the patients with the progressive malignant disease have never been identified. In this context, although mycoplasma-mediated initiation effect in malignant transformation of mammalian cells has not been well demonstrated, mycoplasma-mediated promotional effect in immortalization and malignant transformation of mammalian cells could be clearly demonstrated in the *in vitro* models as described above. Chronic and persistent infections with the unusual prokaryotic microbes known to possess potent promotional effect of malignant transformation *in vitro* could similarly play an important promotional role in the malignant progression of many prostate cancers. Thus, the present finding, if confirmed, would have a highly significant impact and lead to the development of new approaches of preventions, diagnostic tests and therapeutic treatments against prostate cancers.

The concept that bacterial infection could lead to cancer development in human was first proposed in the late nineteenth and early twentieth century. The convincing evidence that linked bacterial infection with the development of various cancers was not demonstrated until the association between *Helicobacter pylori* infection and developments of gastric cancer and mucosa-associated lymphoid tissue (MALT) lymphoma were reported near a hundred years later [17]. Reported evidence of infections by other bacteria species associated with cancer formations in human included *Salmonella typhi* in gallbladder cancer, *Streptococcus bovis* in colon cancer, and *Chlamydia pneumoniae* in lung cancer [18, 19]. Although the present study may be the first demonstration of an association between mycoplasmal infections and cancer development, there were several reports of tumor growth or development of tumor-like lesions in patients with various mycoplasmal infections in lung, pleural sac or pericardium in the past (Table 1). Interestingly, these pseudo-tumors regressed completely following effective antibiotics treatments to eradicate the mycoplasmas. The potent mitogenic effects as well as the potent induction of pro-inflammatory cytokines of the mammalian cells found in the *in vitro* studies could apparently have equally important *in vivo* effects in the infected patients. It is not clear if chronic or prolonged mycoplasmal infections could indeed cause genetic alterations and gene instability of these rapidly proliferating cells in these tumor-like lesions, as clearly documented in many *in vitro* studies of ours and others. Our *in vitro* model systems have clearly demonstrated once these irreversible genetic events occur the malignant transformation of these cells would become permanent, eradication of mycoplasmas can no longer reverse the continuous/unrestrained growth property of these cells, hence formation of true malignant cancer in the infected patients.

**Table 1:** Mycoplasmal infections and development of tumor-like lesions, clinical case reports

Infections	Tumor-like Lesions	Citations
Mycoplasmal pericarditis	Left ventricular hemangioma	Boden WE et al. [20]
M. *pneumoniae* infection	Pleural pseudotumor mesothelial cell proliferation	Abo W et al. [21]
M. *pneumoniae* infection	Pseudotumor of the lung	Park SH et al. [22]

In reviewing the exciting study finding, it is still prudent to note that the high likelihood of both PCR assay and mycoplasma isolation as a result of contamination from various laboratory procedures could often complicate the assessment. This has also been rightly pointed out in another commentary on the current report published in this issue of journal [23]. The study will need to be independently confirmed under a stringent condition. As mentioned earlier, further studies, including solid epidemiological studies and development of animal models, are clearly needed for better assessment of the possible role that, if any, mycoplasmas may be playing in development or progression of prostate cancer. In conclusion, a role for mycoplasmas in any human or animal cancers is still conjectural; however the current report of demonstrating a significant association between *M. hominis* infection and development of prostate cancer has provided an important piece of clinical evidence to support the possibility that chronic infection by the unusual wall-free bacteria could lead to human cancer development. This report should intrigue many more scientists to examine mycoplasma-oncogenesis process and to further investigate potential infectious root in prostate cancer formation or progression.
